# Early union, ‘*disgrasya*’, and prior adversity and disadvantage: pathways to adolescent pregnancy among Filipino youth

**DOI:** 10.1186/s12978-021-01163-2

**Published:** 2021-05-26

**Authors:** Christine Marie Habito, Alison Morgan, Cathy Vaughan

**Affiliations:** 1 Centre for Health Equity, Melbourne School of Population and Global Health, The University of Melbourne, Parkville, Victoria 3010 Australia; 2 Nossal Institute for Global Health, Melbourne School of Population and Global Health, The University of Melbourne, Parkville, Victoria 3010 Australia

**Keywords:** Adolescent pregnancy, Youth, Adolescents, Sexual and reproductive health, Philippines

## Abstract

**Background:**

Few studies explore what it means to be an adolescent parent in the Philippines from the young parents’ perspective. This study sought to improve understanding of how adolescent mothers and young fathers experienced pregnancy in Palawan, Philippines.

**Methods:**

We conducted narrative analysis of 27 semi-structured interviews with 15 Filipino young parents.

**Findings:**

Our findings point to three pathways to adolescent pregnancy differentiated by life circumstances and perceived self-efficacy: through early unions, through ‘*disgrasya*’ (accident) in romantic relationships, and when pregnancy is directly related to adversity and disadvantage. Some young people adopted agentic narratives and had intended pregnancies within early unions. Young people who had unintended pregnancies in romantic relationships recounted constrained choice narratives, taking responsibility for their decisions while emphasising external factors’ influence on their decision-making. Other young mothers described the ways that prior adversity and disadvantage gave rise to unfavourable circumstances—including sexual violence—that led to unintended pregnancy but shared narratives showing how they had reclaimed agency in their lives.

**Conclusion:**

Our findings highlight the need to (1) address underlying poverty and structural inequalities that limit Filipino young people’s life choices and contribute to their pathways to adolescent pregnancy; (2) provide Filipino young people with access to essential sexual and reproductive health information, services, and supplies; and (3) change social norms to rectify gender-based power imbalances and sexual violence.

**Supplementary Information:**

The online version contains supplementary material available at 10.1186/s12978-021-01163-2.

## Plain language summary

This study sought to improve understanding of how adolescent mothers and young fathers experienced adolescent pregnancy in Palawan, Philippines. By analysing the narratives of 15 Filipino young parents, we found three pathways to adolescent pregnancy differentiated by life circumstances and perceived self-efficacy: intended pregnancy through early, cohabiting unions; unintended pregnancy through ‘*disgrasya*’ (accident) in romantic relationships; and unintended pregnancy following prior difficult life circumstances where pregnancy led to reclaiming of agency. Our findings highlight the need to address poverty and inequalities, ensure that Filipino adolescents have access to sexual and reproductive health information, services and supplies, and change social norms that perpetuate gender-based power imbalances and sexual violence.

**Supplementary Information:**

The online version contains supplementary material available at 10.1186/s12978-021-01163-2.

## Background

Around the world, adolescent pregnancy is viewed as a problem largely due to potential health and socioeconomic risks this gives rise to for young mothers and their children. Extensive literature highlights the association between adolescent pregnancy and risks of pregnancy- and childbirth-related morbidity and mortality, and compromised educational and employment prospects, especially in low- and middle-income countries [1–5]. Within these contexts, some factors underlying adolescent pregnancy include early marriage (or union formation) and timing and context of first sex [6]. In addition, structural inequalities—institutionalised social conditions that assign unequal status to some categories of people over others [7] such as age, gender, and class—also make young people more vulnerable to adolescent pregnancy by denying them access to education and sexual and reproductive health (SRH) resources such as SRH information, services, and contraceptive supplies [8].

Despite global and regional trends indicating declining fertility overall, the Philippines is among a few Southeast Asian countries where adolescent fertility rates have either remained stable or increased since the Millennium Development Goals targets were set in 2000 [9]. Childbearing among Filipino adolescents exhibited a generally increasing trend over the last 20 years [10], with data collected in 2017 showing roughly one in 10 Filipinas in this age group was already a mother or pregnant with their first child [11]. Further analyses of Demographic and Health Surveys data found that two out of three women (aged 20–24) who experienced adolescent sexual initiation (i.e. before their 20th birthday) also experienced adolescent pregnancy [12], and that the younger their age at first birth, the more likely they were to experience a repeat pregnancy [13]. However, sexually active adolescent women aged 15–19, whether married or unmarried, also had the highest unmet need for family planning among all women of reproductive age [11]. Consequently, unintended (mistimed and unwanted) births were more likely among adolescent mothers compared to older women, with roughly 30% of births to adolescent mothers reported as unintended [11].

Dominant religious beliefs and values have a strong influence over Filipino adolescents’ SRH and rights [14]. Roughly 80% of Filipinos identify as Roman Catholic [15] and conservative Catholic teachings remain embedded in Filipino social norms, policy and legislation. Catholic doctrine dictates that sex should only occur for procreation within marital unions and as such, premarital adolescent sexuality (especially for young women) and use of modern contraceptives are generally frowned upon [16, 17]. The influence of Catholic teachings is evident in prevailing social norms and sexual double standards that grant men sexual freedom while limiting the sexual and reproductive rights of women [18, 19]. Yet, though conservative ideals and norms remain dominant, progressive Catholics have been dissenting [19] and there is evidence that Filipino women are finding ways to reinterpret restrictive Catholic teachings in ways that allow them to meet their sexual and reproductive needs while maintaining a moral equilibrium [20]. Furthermore, data show that Filipino young people have been gradually adopting more liberal attitudes on sexuality over the years [21].

In 2012, the Responsible Parenthood and Reproductive Health Act (more commonly known as the ‘RH Law’) was signed into law to help improve maternal health and SRH outcomes—especially among poor and marginalised groups—through the provision of essential SRH resources (i.e. information, services, and supplies), emergency obstetric care, and comprehensive sexuality education for adolescents in formal and non-formal education systems, among others [22]. As of this writing, there are other legal measures pending in the Congress of the Philippines that will also affect adolescent SRH in the country, particularly, bills seeking to raise the age of consent from 12 to 16 [23, 24] and provide targeted comprehensive sexuality education and social protections for pregnant and parenting adolescents [25]. However, a significant number of governmental norms and standards continue to impede adolescents’ SRH and rights, among them, the continuing prohibition on legal and safe abortion [14, 26].

Filipino young people’s relationship patterns and social contexts are different to those of previous generations and recent studies affirm that young people’s sexual behaviours do not necessarily align with conservative ideals regarding sexuality [27–28]. National survey data show growing proportions of young people reporting mostly premarital adolescent sexual initiation over a 20-year period [30]. At the same time, the rise of the Internet, digital technology, and social media has been transforming young Filipinos’ social interactions and allowing them to bypass social expectations and norms that do not align with their SRH needs and desires [31]. Studies among Filipino youth have shown that mobile phones and social media are enabling easy access to communication and vast amounts of content, and facilitating online and offline intimacy [32–36]. This has been accompanied by rising cohabitation and the deferral of formal marriage, often due to costs associated with the wedding ceremony and reception and obtaining a marriage license [37–39]. More recent studies have found that cohabitation was more likely among less educated women and men, and argued that the preference for cohabitation among Filipinos was indicative of socioeconomic disadvantage [40, 41].

Conservative social norms regarding premarital adolescent sexuality and contraceptive use contribute to gender-based power differentials within relationships [42, 43] and the stigmatisation of pregnant and parenting adolescents [16]. Dominant depictions of adolescent pregnancy in the Philippines often assign blame to young people’s lack of responsibility [44] and moral decline [45, 46]. However, there has been growing recognition of other important contributing factors, such as poverty and inequality [47–49] and sexual violence [47, 50, 51]. For example, a study in Cebu City, Philippines found that 72% of young women experienced unwanted sexual initiation [52], while another cited that Filipino adolescents’ intimate partners commonly used verbal pressure to pursue coercive sex [53].

Indeed, national statistics show that women with lower levels of education and from resource-poor backgrounds are more likely than their wealthier and more educated counterparts to have begun childbearing as adolescents [11]. Government leaders point to early union formation on account of pregnancy as the main reason for school ‘dropout’ among Filipino youth [54], thereby limiting their lifetime wage-earning potential and costing the country billions in lost gross domestic product [55, 56]. However, others emphasise that sometimes, poverty and other adverse life events forces a young person to abandon their education which subsequently results in adolescent pregnancy, highlighting how adolescent pregnancy may arise when young people—especially girls—have limited educational and employment opportunities [42, 57]. Furthermore, some women pointed to having little or no choice but to accept sex when male partners were manipulative or coercive [20]. Recent vital statistics data suggest that most births to adolescent women (i.e. 10–19 years old) were fathered by older (non-adolescent) men [50, 58], indicating that power differentials within relationships may be increasing the risk of sexual coercion and abuse. Studies have found that Filipino women from lower wealth quintiles were more likely than women from higher wealth quintiles to experience forced first sex [59] and women who experienced unwanted first sex were more likely to go on to experience unintended pregnancy than women whose first sex was wanted [52]. These studies suggest that the circumstances leading up to Filipino adolescents’ experiences of pregnancy are varied and complex.

Qualitative research can serve as an important counterbalance for dominant narratives that are often based on quantitative analyses and are limited to describing associations between a range of determinants and adolescent pregnancy outcomes [60, 61]. Studies from other settings have focused on young mothers’ narratives of their lived experiences and sought to offer more balanced views of pregnant and parenting adolescents’ realities by presenting positive along with negative experiences and outcomes [62, 63]. Deirdre Kelly discusses four discourses of adolescent mothers commonly presented in mainstream media [64]. These include the ‘wrong girl’ frame which blames the young woman and her ‘dysfunctional’ upbringing for an adolescent pregnancy; the ‘wrong family’ frame which laments the breakdown of ‘traditional’ (heterosexual, two-parent families) families and government spending on young, single parents; the ‘wrong society’ frame which argues that adolescent pregnancy is a marker of structural inequalities within society; and the ‘stigma is wrong’ frame which represents the viewpoints of the young parents who believe that they should not be stigmatised as they are capable of being good parents to their children. These discourses can be useful for analysing and rethinking common assumptions made about adolescent pregnancy.

This study is a component of a larger doctoral research project that aimed to gain a deeper understanding of factors associated with adolescent pregnancy and parenthood among Filipino young people. We explored experiences of adolescent pregnancy from the perspective of pregnant and parenting Filipino youth and examined them guided by the four discourses outlined by Deirdre Kelly [64]. We believe that it is important to consider young parents’ motivations and contexts to better understand how they interpret their experiences of adolescent pregnancy amid the very real impacts of poverty and structural inequalities on their lives. In doing so, we hope to highlight their unique circumstances and needs so that they can be provided with more meaningful health services and forms of support. This paper presents our findings pertaining to Filipino young people’s pathways to adolescent pregnancy through the narratives of young women who became mothers during adolescence, as well as those of young men who became fathers with adolescent partners.

## Methods

### Study setting

We conducted this study in Puerto Princesa City in the Province of Palawan, Philippines. Puerto Princesa is made up of 19 urban and 47 rural *barangays* (villages) [65]. The majority (80%) of the population of Puerto Princesa identify as Roman Catholic and about one-third are young people (aged 10–24) [66]. Between 2010 and 2015, Puerto Princesa was experiencing rapid population growth alongside economic and infrastructure development, largely due to the growth of the tourism industry [67]. However, this growth and development was most visible in the central business district, outside of which there were large stretches of sparsely populated forested and agricultural lands where agriculture and fisheries remained the dominant source of livelihood [68, 69].

### Study design

This study used semi-structured, individual interviews with Filipino young people to inquire about their lived experiences of pregnancy and parenting, including their relationship contexts leading up to pregnancy; feelings on first learning of the pregnancy; hopes and fears for their child and themselves; and the kinds of services and support they believed were needed by young people and young parents in their community. We used a semi-structured interview guide (Additional file 1) but also allowed some flexibility during interviews to discuss other related topics that were important to the participants. The study received ethical clearance from the Human Ethics Sub-Committee of the University of Melbourne (ethics ID 1851023.1), and the De La Salle University Research Ethics Review Committee (ethics ID EXT-008.2017-2018.T2).

### Recruitment

We partnered with a local non-government organisation (NGO) that provides health services to women and young people. Participants were recruited at the partner NGO’s clinical services and through referral by some of the NGO’s staff and volunteers. We invited 24 adolescent women (aged 15–19 who had experienced pregnancy or were pregnant at the time of data collection, as well as seven young men (aged 15–24) who had an adolescent partner/girlfriend (aged 15–19) who was pregnant at the time of data collection or had experienced pregnancy in the last few years. To avoid potentially causing conflict between partners, we did not invite participants who were partnered with each other to both participate (i.e. only one person from a partnership was invited). The first author approached each prospective participant in person; gave a brief description of the study, its purpose, and what participation entailed; gave the participant time to ask questions; and provided a copy of the plain language statement for the participant to keep. We asked prospective participants for their preferred method of follow-up, and at least 24 h after the initial meeting, confirmed in-person, or via call or text message whether they were willing to participate in the study. Sixteen of the prospective participants did not respond to our efforts to confirm their interest or expressed that they were too busy to participate. In all, ten adolescent mothers and five young fathers agreed to participate in our study. All the participants were from low- to lower-middle income backgrounds—their family members and partners were either unemployed or subsistence wage-earners working casual jobs in the fisheries, construction, tourism, and service industries. Twelve participants were from urban or peri-urban communities, and three young women were from rural communities.

### Data collection

Data used in this study were collected between August 2018 and March 2019. All data collection activities were conducted in Tagalog by the first author who is a female PhD candidate with a background in development studies and research, a native speaker of Tagalog, and fluent in English. Before each interview, the first author endeavoured to establish rapport and trust with the participant by reassuring them that she was neutral to the subject and that the interview was a safe space for them to share their thoughts and experiences. Fifteen initial interviews were conducted (each lasting 20–60 min). Interviews were done at participants’ homes or in quiet spaces in public locations where participants felt comfortable and privacy could be assured. In three interviews conducted at participants’ homes, some family members were present, and privacy could not be assured. In these cases, the first author proceeded with the interview but also returned at another time to conduct another interview when privacy could be assured. All interviews were audio-recorded with the informed consent of each participant. The first author made note of recurrent themes and took field notes. We conducted 12 follow-up interviews (each lasting 12–90 min) with participants who were willing and able to continue participation in the months following the initial interview. Though we sought to arrange follow-up interviews with all participants, some were not available due to childcare, work, or other responsibilities. The first author reviewed all audio recordings of the initial interviews before scheduling follow-up interviews to ensure that anything unclear could be clarified with the participant. Each participant received a cash incentive of PhP100 (~ US$2) at the end of every interview and was reimbursed any costs associated with travelling to the interviews in instances where this was necessary.

### Data analysis

The first author transcribed each audio recording verbatim and translated relevant excerpts to English. We used QSR International’s NVivo 12 to organise the interview transcripts and to support coding and analysis. We assigned pseudonyms to each participant and these are used in quotations in the Findings section. Original language versions (in Tagalog/Taglish) of all quotations cited are provided in Additional file 2.

We used a narrative approach as described by Clandinin [70] when analysing young people’s lived experiences of adolescent pregnancy. Narrative analysis is an appropriate approach for understanding how people process their life experiences (in this case, adolescent pregnancy) and what is important to them [71]. As in Riley and Hawe [71], we took note of how our participants told their stories and constructed their sentences; what their assessments were of specific events in their lives leading up to their pregnancies and why they felt that way; who the ‘supporting cast’ were that they included and excluded in their narratives; the context within which their story was being told (and our role in it); and what the point of their story seemed to be about. We also paid attention to the structure of participants’ narratives, noting whether they depicted themselves in agentic positions—taking responsibility for their circumstances and having a plan for themselves—in victim positions that focus on the tragic elements of their life experiences and resigning their future to fate, or a combination of the two. Based on the details participants shared through their narratives, we mapped each participant’s trajectory to adolescent pregnancy, taking note of pivotal life events, contributing factors, and depictions of choice and control included in their stories, and identified commonalities to come up with pathways to adolescent pregnancy. In presenting our participants’ narratives, we remain mindful of the importance of not ‘finalising’ the stories of participants for them, giving them room to change the direction of their stories and their lives [72].

### Findings

Our data revealed three pathways to adolescent pregnancy. The pathways are not mutually exclusive—there are some elements of participants’ stories that overlap with those also found in other pathways. However, each pathway was differentiated by participants’ specific life circumstances and their perceived self-efficacy—what they believe they can achieve through their actions [73]—under those circumstances, including contextual factors preceding conception, perceived control (or lack of it) over their past, present, and future, and their feelings about their experiences. While the young people described pregnancies occurring in diverse circumstances, our participants’ narratives can be clustered into three main pathways to adolescent pregnancy: (1) Early union as a life choice; (2) ‘*Disgrasya’* (accident) in romantic relationships; and (3) Prior adversity and disadvantage. These pathways highlight important life events that participants included in their narratives and the ways in which they linked those events to their experiences of pregnancy and parenthood.

### Pathway 1: Early union as a life choice

*We'd waited for years. We were going on three [years together], only then did I get pregnant. ... I was very happy because he wanted a child, I also wanted a child. We had the same joy. ... [Our parents] were happy too. Very happy, because of course, they wanted a grandchild. (Belle, age 19, seven months pregnant)*

Pathway 1 aligns with the trajectory of young people who chose to begin family life in cohabiting unions with the support of their families and then had a planned pregnancy in the context of that union. For three young women in this study, pregnancy and parenthood were expected, valued milestones in their transition to family life.

Belle, 19, moved into her partners’ family home when she was 16 years old and stopped attending school around the same time. Elements of her narrative indicated that her natal family’s financial difficulties likely influenced her decision to move in with her partner early. Belle was aware that legal marriage was still preferred over informal unions in her community, yet she seemed perfectly happy in her live-in relationship and role as partner and mother-to-be. She recounted experiences of not having enough or any food to eat and worried about whether her partner’s income as a fisherman would be enough to support their future family. However, this did not detract from her excitement about the birth of her baby or dampen her hopes of a better future. She was determined to return to school eventually to finish her education and become a teacher so she could help her partner to earn income for their family. Though Belle talked about the difficult aspects of her life that were beyond her control and represented disadvantage, she used these experiences to imagine the ‘good life’ that she wanted for her family while remaining cognisant that ‘life is difficult’.

One young mother demonstrated considerable agency in her decisions leading up to union and pregnancy. For Diane, 19, moving in with her boyfriend at age 17 was at her parents’ suggestion, but she did not want to get pregnant immediately, and her parents supported her decision. They provided her with the consent that she needed to access free contraceptive pills at the local health centre.

*[My mama gave consent] because I told her that I didn't want to have a child yet. … Papa, too. I said, ‘I don't want it [to get pregnant] yet, Pa, even though I married early, I don't want to get pregnant immediately.’ (Diane, age 19, four months pregnant)*

For 2 years, Diane took pills and opted to not tell her partner because she was ‘*nahihiya*’ (embarrassed; ashamed) to admit to him that she did not want a baby yet, even though he and his family did. She experienced pressure from extended family members to bear a child to secure her relationship but remained steadfast in her decision to wait. Eventually, when she felt ready for parenthood and became convinced that her partner was too, she stopped taking the pills. She was happy about her pregnancy but admitted that part of her motivation was to accommodate her partner’s elderly parents’ request to see a grandchild from them before they died. Delaying pregnancy allowed Diane to complete a post-high school vocational course before she became a mother, so she was confident that her qualification would allow her to find employment opportunities when she was ready to work. With the support of her parents, Diane met her educational goals, took control of her reproductive decisions, and made concrete plans for her future.

### Pathway 2: ‘Disgrasya’ (accident) in romantic relationships

*I didn't know I was already pregnant then. … I took a pregnancy test, then I was pregnant. I said [to my boyfriend], ‘This is already here, it would be a big sin if we abort.’ … It was only when we found out that I was pregnant [that] he said he could do it [provide for us]. … [I was having] second thoughts … because I am still a baby, then I already have a baby. (Giselle, age 15, five months pregnant)*

Young parents who followed the second pathway had unintended pregnancies in the context of romantic relationships, largely when couples had been exclusively dating for at least a few months. The term ‘*disgrasya’* (accident) was often used by study participants to refer to unintended pregnancies. Despite their awareness that sexual activity before marriage was not socially accepted, some participants explicitly linked their unintended pregnancy to consensual sex that had been motivated by curiosity and romantic love. For example, Noel was 16 years old and in a romantic relationship with his girlfriend when they had sex out of curiosity and had an unintended pregnancy. Similarly, 16-year-old Jenny admitted thinking that maybe she wanted to get pregnant but changed her mind upon learning of her pregnancy.

*That love-love – it’s like you think that you want to get pregnant. But when I got pregnant, that’s when I realised that it was very difficult, especially when your partner leaves you. (Jenny, age 16, eight months pregnant)*

Although the resulting pregnancies were unintended, these young people described sex as having been sporadic and opportunistic but wanted; it was an expression of emotional intimacy with a trusted partner, albeit with an unexpected outcome.

Learning of their pregnancy, figuring out what to do, and breaking the news to their parents, relatives, and friends was a stressful ordeal for the young people who aligned with this pathway. They feared being scolded, physically hurt, disowned, and/or kicked out of the house by their parents. Nevertheless, for most of them, family and friends were quick to ‘accept’ (or become resigned to) the pregnancy news and from then on, became the young parents’ primary sources of support. The ‘acceptance’ of family and friends gave way to feelings of relief and allowed the young parents to feel excited about the birth of their baby while remaining mindful of the challenges ahead.

Initially, they associated the pregnancy with external factors or circumstances that they perceived to be beyond their control, such as partners initiating sex or boyfriends failing to practice withdrawal; not having/being given SRH information and supplies; and, as in the case of Leo, 18, wanting to escape problematic situations at home.

*I was a working student, and every morning, my parents were noisy [fighting]. … I felt like I was alone. … I was stubborn, too, because my girlfriend – I would always go to her. Of course, I was getting irritated [with my parents] already. Then I thought, ‘What if I just live on my own?’ That was what was on my mind during that time. Then, that was what happened – [we] bore fruit. (Leo, age 18, 17-year-old partner was seven months pregnant)*

Sixteen-year-old Elaine looked back at her unintended pregnancy as ‘God’s will’ for her and a ‘blessing’:

*At first, [I thought it was] probably God's will for me. Because if it wasn't, why would he give this to me, right? So, I thought, ‘God probably gave this to me, this kind of blessing.’ (Elaine, age 16, baby was 1 month old)*

These young parents recognised that their decisions led to unintended pregnancies, but it was important to them to highlight in their narratives that specific life circumstances were affecting their decision-making, and they were not fully informed or equipped at the time. Some young mothers felt they lacked knowledge about and access to contraceptives. For instance, one young mother did not know about women’s contraceptive choices when she became sexually active because she believed that contraceptives were only for married people. Meanwhile, young mothers and fathers downplayed the possibility of pregnancy based on myths and misinformation about sex and reproduction, especially the common belief that withdrawal was an effective way to prevent pregnancy. Young fathers acknowledged knowing about contraceptives but that they had not thought contraception was important and had not used modern methods consistently (if at all). Oscar, 23, recalled giving little thought to pregnancy even though he and his then-17-year-old girlfriend were aware of the possibility.

*It didn't become important [to us] during those times. Because when you are young, you just want to enjoy, right? It's like, we didn't think of that. ‘Ay, that won't happen. Not like that. That's okay.’ … That was probably what was lacking with us. (Oscar, age 23, became a father at age 22 with his 17-year-old girlfriend)*

While taking responsibility for their unintended pregnancies, some of these young parents believed they would have made different choices if they had the resources and support that they needed and a better understanding of how unintended pregnancy would change their lives.

### Pathway 3: prior adversity and disadvantage

*At first, I stopped [attending school] because I took care of my sibling. … That’s why I became careless with myself. ... My thinking back then was, ‘It's always me,’ because I was the only one doing anything in the house. … I was the only one they [my parents] could depend on, I looked after my sibling, I cleaned, I did this and that. … I also wanted to enjoy [myself]. This [pregnancy] is what happened from my enjoyment. (Indy, age 15, baby was four months old)*

In the third group of narratives, prior adversity and disadvantage gave rise to pivotal events in the lives of young mothers that were key antecedents of an unintended pregnancy. All the young parents who participated in this study were from resource-poor backgrounds. However, unlike the young people whose stories mapped to pathways 1 and 2, in the third cluster of narratives, poverty and ill-health were prominent factors that led to young women discontinuing their education and this was something over which they felt they had no control. Leaving school introduced changes to these young women’s life circumstances that increased the risk of unintended pregnancy.

For some young women, their families’ lack of resources meant having no choice but to leave school to put their family’s needs before their own, and it was while they were out of school that they became pregnant. Candy, 16, left school when she was 15 because her family struggled to cover her school expenses. Candy met her current partner through text messaging while she was working as a *kasambahay* (domestic helper). Three months after meeting in person for the first time, they decided to move in together in his rural hometown. All Candy wanted at the time was to always be with her partner; she had not intended to start a family yet. She described taking contraceptive pills but not taking them every day and attributed her pregnancy to this. Although her pregnancy was unintended, Candy was quick to accept it; she decided that she was happy about it and looked forward to having a family of her own. For Candy, being in a stable cohabiting relationship allowed her to embrace the idea of becoming a mother sooner than she intended and adopt a positive outlook in her narrative.

For some young women, adversity took the form of sexual violence. Three young women in this study experienced sexual violence leading up to their pregnancies. Aya, 19, had experienced ill-health, the death of her mother, and had dropped out of vocational school. She was also subject to sexual violence. Aya’s first sex with her boyfriend was forced. Although she reported that their subsequent sexual encounters were consensual, she admitted contemplating suicide when she learned of her first unintended pregnancy.

*It came to a point where I thought about killing myself. ... Because there was so much, so much. Also, the things people were saying, it's like they were adding to it. ... I just thought about it, but I didn't do it because I was afraid. (Aya, age 19, first pregnancy at age 16, second pregnancy at age 18)(Aya, age 19, first pregnancy at age 16, second pregnancy at age 18)*

Aya saw her first pregnancy through but her baby died of an illness less than two months after birth. Having no work and no money to return to school, Aya was housebound; it was then that she was sexually abused by her father. Aya had no one she could talk to about it, so she coped by spending as much time as she could with her *barkada* (peer group) to stay out of the house. When Aya had a second unintended pregnancy with a new boyfriend, she felt it was ‘okay’ to start a family; she saw it as her way out of her troubled home situation. Aya’s first sex, pregnancy, and transition to family life happened before she felt she was ready. However, she prioritised her responsibilities as a mother and looked forward to a time when her child could be left in someone else’s care so she could try to find a job.

Two young mothers in this study became pregnant because of rape. Helena, 16, had been in her first romantic relationship for a few months when she experienced forced first sex. She stayed in the relationship despite it, and months later, her boyfriend raped her again, which then resulted in her pregnancy.

*The second time [my boyfriend forced me to have sex], I just thought, ‘We're approaching one year together already...’ I thought – because normally, it happens between a boyfriend-girlfriend couple, right? ... After that, I just didn't say anything. … I just let him do what he wanted because I thought, ‘It already happened the first time.’ (Helena, age 17 at follow-up, baby was four months old)*

Shortly after, Helena ended her relationship with her baby’s father. Yet, Helena recalled how happy she felt when she had her first ultrasound and heard her baby’s heartbeat. Helena was intent on finishing her education and finding work after giving birth. After her traumatic first relationship experience, she no longer had any ambitions of having boyfriends like other girls her age. She saw the pragmatic choice as being to prepare herself for the reality of impending childbirth and single motherhood. Like the other young women whose stories aligned with this pathway, Helena positioned herself as having been a victim in the past but was taking charge of her present and future by focusing on what she could realistically accomplish given her circumstances and available resources. This allowed her to reclaim control over her future by changing her narrative from that of a victim to an active agent.

## Discussion

Our participants’ accounts of their experiences of adolescent pregnancy illustrate nuanced and complex pathways to parenthood. The key finding of our study was that there is no single track to adolescent pregnancy among Filipino youth. Young parents’ narrative structures varied according to their past experiences, their intentions, how they interpreted and felt about their experiences, and their outlook for the future.

Young parents belonging to pathway 1 told agentic narratives about their planned adolescent pregnancies within the context of informal/cohabiting unions. These young parents portrayed their unions as their decision—rational choices made in light of financial, educational, and social acceptability considerations, and the support of both sides of the family. As in Deirdre Kelly’s ‘stigma is wrong’ discourse [64], young mothers in pathway 1 drew on ‘themes of empowerment, rejecting messages that portray them as victims’. In contemporary Philippines, informal cohabiting unions have been increasing in place of formal marriage [40]—one in four 15–24-year-olds were either living with their partner as if married or had ever been married [11]. The narratives of young people who followed this pathway demonstrated that starting family life early can be an alternative path to self-fulfilment, especially when there are barriers to other age-appropriate life goals (e.g. education, employment) [42, 57]. Within these early unions, pregnancy was not only expected but wanted and celebrated.

Through the support of her parents, one young mother in our study was able to use modern contraceptives to assert her reproductive preferences, achieve her educational goals, and plan for her family’s future according to her needs and aspirations. Hers was by far the most agentic narrative we encountered. Yet, this young mother was aware of the social expectation for women to begin childbearing as soon as possible after union formation and instead of openly challenging it, chose to quietly resist. She demonstrated that given the necessary parental support, Filipino young people recognise self-efficacy and exercise agency in their relationships and plan for their futures. However, her narrative also draws attention to the social pressures that Filipino young women continue to face regarding their reproductive choices—in this case, having a baby to secure a partnership and to accommodate family expectations to bear children [74, 75].

The stories of participants who aligned with pathway 2 revealed narratives of constrained choice as opposed to dominant narratives that portray young parents as unwitting victims of youthful emotions and impulses. In their narratives, participants reproduced elements of what Deirdre Kelly referred to as ‘wrong girl’ and ‘wrong society’ discourses [64] which assign responsibility for unintended pregnancy to the young people but also call out how adults and broader society have failed to provide for their needs. These young parents strategically framed their experiences of unintended pregnancy by elaborating on external (social) factors and actors beyond their control, highlighting that their decisions were their own but made without critical resources and support.

Though these accounts aligned in part to dominant narratives that link young people’s transitions to parenthood to *‘disgrasya’* (accident), they also reveal important considerations not always included in mainstream characterisations of ‘teen parents.’ First, for some young people, though pregnancy was not expected, sex was wanted. They had romantic feelings for their partners, were curious about sex, and wanted to be sexually intimate even amid known social disapproval of premarital sex [27, 29]. Second, some pregnancy outcomes were linked to external factors over which young people felt they did not have control, such as their partners’ sexual desires or contraceptive behaviour, their access to essential SRH information and supplies, conflict in their home environments, and fate or God’s will. For young women, decisions on whether and when to have sex or use contraception were subject to the balance of power between them and their boyfriends [27, 42, 76]. Third, young parents were aware that they did not know enough about or give enough importance to contraception, and though they took responsibility for their unintended pregnancies, they were also aware that they were not fully equipped to prevent them. Analysis of national survey data found that the most common reason cited by sexually active, unmarried Filipinas for not using contraceptives was that they were ‘not married’ [77] which aligns with our participants’ beliefs that they were unlikely to get pregnant or that contraceptives were only for married people. Lastly, young parents very much valued and depended on the acceptance and support of their family and friends, especially when their relationships with their partners dissolved. This is consistent with studies with Filipino youth which found that partner and family support were important determinants of whether unintended pregnancy resulted in birth or induced abortion [78, 79].

For participants who followed pathway 2, drawing attention to external factors allowed them to distribute accountability among the supporting cast in their stories (i.e. their partners, families, friends) while still taking responsibility for their decisions. Our findings from this pathway underscore the need to recognise young people as emerging adults with needs, emotions, and desires; consider the role of power relations between intimate partners in sexual decision-making and contraceptive behaviour; address myths and misinformation that contribute to low pregnancy risk perception and contraceptive mis- and non-use; and encourage young people’s families and friends to adopt more accepting, supportive attitudes in the event of unintended adolescent pregnancy.

The third pathway affirmed how structural inequalities can create what Deirdre Kelly referred to as the ‘wrong society’ [64] where young people become more likely to experience unintended pregnancy. As all our participants were young people from resource-poor backgrounds where conservative social norms perpetuated gender-based inequalities, structural inequalities pertaining to age, wealth, and gender were present across pathways. However, for young parents belonging to pathway 3, leaving school because of a confluence of poverty, ill-health, and other forms of adversity and disadvantage (e.g. non-inclusive school policy, lack of childcare support to indigent families) was a salient aspect in their pathway to unintended pregnancy. The storylines of young mothers belonging to pathway 3 demonstrated how structural inequalities can force families to sacrifice a young person’s education due to lack of options or constrain their ability to assert their sexual and reproductive preferences in their relationships, thereby influencing their life trajectories. They shared narratives where they had been victims, recounting their historical experiences of adversity and disadvantage, but transitioned to agentic narratives in discussing the present and future. Unlike participants belonging to pathway 1 who viewed early union and pregnancy as deliberate and desirable life choices, young parents aligned with pathway 3 perceived the adversity and disadvantage that they experienced as beyond their control and as important elements in their narratives of unintended pregnancy. Like their counterparts in pathway 1, however, they were also cognisant of the opportunities and possibilities still available to them and worked within their constraints to find meaning and purpose in their lives.

Recently, policymakers have turned their attention to the role of sexual abuse in adolescent pregnancies in the Philippines, noting that sexual violence in the country is high [50]. For three of the ten young mothers we interviewed, their unintended pregnancies were preceded directly or indirectly by experiences of sexual violence. Yet, they were determined to move forward with their lives as they saw no other choice and had a child for which to provide and care. These young mothers included tragic elements in their stories of the past, but they resisted being defined by their experiences of violence and were reclaiming narrative agency [80] in the way they discussed their current situation and outlook for the future. They showed that even after enduring sexual abuse, young mothers can demonstrate an emerging resilience as a ‘choice of the necessary’ [62, 81]. However, the social structures that perpetuate gender-based inequality and sexual violence need to be addressed if SRH programmes seeking to curb unintended adolescent pregnancies are to result in better health and life outcomes for Filipino young women and men.

### Limitations

We note three main limitations to our study. Our use of non-random sampling and a small number of participants limits the generalizability of our findings. Nonetheless, there is value in re-evaluating dominant narratives about young parents, especially if this facilitates a better understanding of who young parents are and what they need [80]. Second, young mothers were recruited at health clinics offering free prenatal check-ups for pregnant women from resource-poor communities, as well as through referral of the partner NGO’s staff and volunteers. The fact that the young mothers were demonstrating health-seeking behaviour could mean that our participants were more likely to have resolved to care for their baby regardless of their pregnancy intention, and thus, be more willing or better able to see their pregnancy in a positive light. Also, our sample does not capture the experiences of young people who opted to abort their pregnancies. Abortion remains illegal in the Philippines, but as our study participants pointed out, community members were well-aware of how to access clandestine abortion services when needed. Although we were able to capture rich, nuanced narratives from our participants who chose to see their pregnancies through, there would certainly be merit in research with young people for whom pregnancy termination was the ‘responsible choice’ [82]. Finally, our participants were at different stages of pregnancy and parenthood and were asked to reflect on their lived experience to date, which could have introduced bias in their responses. It is possible that our participants may have forgotten certain aspects of their experiences, or that their assessments of their circumstances and feelings had since evolved. Also, our participants were aware that sex and pregnancy before marriage are generally frowned upon in Filipino society, so it was also possible that they tailored or tempered their narratives to better align with what they perceived to be socially acceptable or desirable.

## Conclusion

We sought to explore Filipino young people’s experiences of adolescent pregnancy in the hope of improving understanding of who young parents are and what they need through their narratives. We found that there is no single track to early parenthood among Filipino youth, and each young person’s trajectory to adolescent pregnancy is differentiated by specific combinations of adversities and by their perceived self-efficacy given their circumstances. Programmes and policy need to be grounded in the realities of the target population to be truly effective, and thus, young people’s narratives of their experiences of pregnancy are valuable inputs to interventions seeking to improve adolescent SRH outcomes. Our findings highlight the need to address poverty and structural inequalities that limit young people’s life choices and contribute to their trajectories to unintended adolescent pregnancy; provide Filipino adolescents with access to SRH information, services, and supplies in enabling environment; and challenge social norms that perpetuate gender-based power differentials and sexual violence.

## Supplementary Information

**Additional file 1.** Interview guide for pregnant and parenting young people.

**Additional file 2.** Original language versions (Tagalog/Taglish) and English translations of interview transcripts.

## Data Availability

The datasets generated and analysed during the current study are not publicly available but are available from the corresponding author on reasonable request.

## Abbreviations

DHS: Demographic and Health Surveys
NGO: Non-Government Organisation
SRH: Sexual and reproductive health
