# Supplementary material for: Early union, ‘disgrasya’, and prior adversity and disadvantage: pathways to adolescent pregnancy among Filipino youth
Source: Reprod Health. 2021 May 26;18:107. doi: 10.1186/s12978-021-01163-2 (PMC8157620; doi:10.1186/s12978-021-01163-2)
Supplement: Supplementary file 1 — Additional file 1. Interview guide for pregnant and parenting young people. [file 12978_2021_1163_MOESM1_ESM.docx]

Additional file 1

# **Interview guide for pregnant and parenting young people**

1. How did you feel when you first found out that you were (your partner was) pregnant?

- Who did you tell first? Why?
- How did your partner feel about/react to the news? How did this make you feel?
- How did your family and friends feel about/react to the news? Other relatives?

1. How do you feel about your (partner’s) pregnancy now?

- If it has changed compared to how they felt when they first found out, why? What changed?

1. What is it like to be a young parent or parent-to-be in Puerto Princesa?

- Thinking about your life before you found out you were (your partner was) pregnant, what aspects of your day-to-day life have stayed the same? What aspects have changed?
- How do you feel about these?

1. What are your hopes and worries/fears for your child, and for your future as an individual, and as (you become) a parent?

- Why do you feel this way?

1. What kinds of sexual and reproductive health services do you think young people in your community need?
2. What kinds of support do you think young parents and parents-to-be need?

- Are these available in your community?
- If yes, are you able to access them? Why/why not?
- If no, what would be the closest alternative?
