# Supplementary material for: Early union, ‘disgrasya’, and prior adversity and disadvantage: pathways to adolescent pregnancy among Filipino youth
Source: Reprod Health. 2021 May 26;18:107. doi: 10.1186/s12978-021-01163-2 (PMC8157620; doi:10.1186/s12978-021-01163-2)
Supplement: Supplementary file 2 — Additional file 2. Original language versions (Tagalog/Taglish) and English translations of interview transcripts. [file 12978_2021_1163_MOESM2_ESM.docx]

Additional file 2

# Original language versions (Tagalog/Taglish) and English translations of interview transcripts

| **Original language version (Tagalog/Taglish)** | **English translation** |
| --- | --- |
| Ilang years po naming hinintay. Magtatatlo[ng taon] na po kami, bago lang po ako nabuntis. … Tuwang-tuwa din po [ako], kasi gusto niya po magkaron ng anak, gusto ko rin po ng anak. Pareho po kami ng saya. … Masaya rin po [ang mga magulang namin]. Tuwang-tuwa, kasi po syempre, gusto rin po nilang magkaron ng apo.  Belle, age 19 | We'd waited for years. We were going on three [years together], only then did I get pregnant. ... I was very happy, because he wanted a child, I also wanted a child. We had the same joy. ... [Our parents] were happy too. Very happy, because of course, they wanted a grandchild.  Belle, age 19 |
| Mama ko lang [ang nagbigay ng consent] kasi, nagsabi rin kasi ako na ayaw ko pa mag-anak. … Pati si Papa. Sabi ko, ‘Ayaw ko pa kasi, Pa, mag-ano, kahit na maaga akong … nag-asawa, ayaw ko muna magbuntis kaagad-agad.’  Diane, age 19 | [My mama gave consent] because I told her that I didn't want to have a child yet. … Papa, too. I said, ‘I don't want it [to get pregnant] yet, Pa, even though I married early, I don't want to get pregnant immediately.’  Diane, age 19 |
| Hindi ko rin kasi alam na buntis na ako nun. … Nag-PT (pregnancy test) po ako, tapos buntis, buntis po ako. Yun sabi ko [sa boyfriend ko], ‘Nandito na ‘to, malaking kasalanan naman pag pinalaglag pa.’ … Tapos saka lang, nung nalaman kong buntis na ako, kaya niya naman daw. … Nagdadalawang-isip [ako]. … kasi baby pa ako, tapos may baby [na] ako.  Giselle, age 15 | I didn't know I was already pregnant then. … I took a pregnancy test, then I was pregnant. I said [to my boyfriend], ‘This is already here, it would be a big sin if we abort.’ … It was only when we found out that I was pregnant [that] he said he could do it [provide for us]. … [I was having] second thoughts … because I am still a baby, then I already have a baby.  Giselle, age 15 |
| [‘Yang] mga love-love na yan – nakaka-ano [isip] ka na, parang gusto mong mabuntis. Pero yung nabuntis po ko, dun ko na-realize na sobrang hirap kasi, lalo na yung iwan ka rin po ng partner [mo].  Jenny, age 16 | That love-love – it’s like you think that you want to get pregnant. But when I got pregnant, that’s when I realized that it was very difficult, especially when your partner leaves you.  Jenny, age 16 |
| Nagwo-working student po ako, tapos kumbaga, tuwing umaga na lang, ang ingay po kasi ng magulang ko. Kumbaga, ano, parang, ako ganun na mag-isip, parang, para hindi ko na sila kasama. … Nararamdaman ko, parang nag-iisa na lang ako. … Tapos yun, matigas din naman po yung ulo ko, dahil nga po sa girlfriend ko – lagi ko pong pinupuntahan. Syempre, naririndi rin po ako [sa magulang ko]. Tapos yun, naisip ko, ‘Ganito na lang kaya gawin ko, bumukod na lang kaya ako?’ Ayun ang nasa utak ko, nung time na yun. Tapos yun na po yung nangyari – nagbunga [kami].  Leo, age 18 | I was a working student, and every morning, my parents were noisy [fighting]. … I felt like I was alone. … Then, I was stubborn, too, because my girlfriend – I would always go to her. Of course, I was getting irritated [with my parents] already. Then I thought, ‘What if I just live on my own?’ That was what was on my mind during that time. Then, that was what happened – [we] bore fruit.  Leo, age 18 |
| Nung una po talaga, [naisip ko] siguro will talaga sakin ni God. Kasi kung hindi naman, bakit niya naman ako bibigyan, ‘di ba po? So, yun po, sabi ko, ‘Siguro bigay talaga ni God sakin ‘to, yung ganitong blessing.’  Elaine, age 16 | At first, [I thought it was] probably God's will for me. Because if it wasn't, why would he give this to me, right? So, I thought, ‘God probably gave this to me, this kind of blessing.’  Elaine, age 16 |
| Siguro hindi siya naging importante [sa amin] nung mga time na yun. Kasi yung, pag bata ka, gusto mong mag-enjoy eh, ‘di ba? Parang, hindi namin inisip yun. ‘Ay hindi yan ano, lalabas. Hindi ganun. Okay lang yan.’ … So yun, yun ang siguro kakulangan na nangyari samin.  Oscar, age 23 | It didn't become important [to us] during those times. Because when you are young, you just want to enjoy, right? It's like, we didn't think of that. ‘Ay, that won't happen. Not like that. That's okay.’ … That was probably what was lacking with us.  Oscar, age 23 |
| Nung una, nag-stop ako [pumasok sa school] kasi ako yung nag-alaga sa kapatid ko. … Kasi parang hindi naman ako sumunod sa kanila, parang, kaya ako nagpabaya-baya. Nawala na rin kasi ako sa ano na, kasi parang, ano ko sa isip ko dati, puros ako na lang. Kasi dati ako lang kumikilos sa bahay. … Ako lang naaasahan, ako nagbabantay sa kapatid ko, ako naglilinis, ako ganito. … Gusto ko din mag-enjoy-enjoy. [Ito ang] nangyari sa kaka-enjoy ko. Pero hindi ko naman siya pinagsisihan.  Indy, age 15 | At first, I stopped [attending school] because I took care of my sibling. … I didn't obey them [my parents], that's why I became careless with myself. ... My thinking back then was, ‘It's always me,’ because I was the only one doing anything in the house. … I was the only one they [my parents] could depend on, I looked after my sibling, I cleaned, I did this and that. … I also wanted to enjoy [myself]. This [pregnancy] is what happened from my enjoyment.  Indy, age 15 |
| Dumating sa punto na ano, na naisip kong mag-ano, pakamatay, ganun. … Kasi ang dami, ang dami. Saka po yung mga salita-salita [ng ibang tao], parang humahalo po. … Naisip ko lang rin, pero hindi ko naman po ginawa kasi takot po ako.  Aya, age 19 | It came to a point where I thought about killing myself. ... Because there was so much, so much. Also, the things people were saying, it's like they were adding to it. ... I just thought about it, but I didn't do it because I was afraid.  Aya, age 19 |
| Nung second time [na pinilit niya akong mag-sex], sabi ko na lang [sa sarili ko], ‘Pa-one year na din [kami]...’ Sabi ko [sa sarili ko] – kasi normally ‘di ba, nangyayari naman yun sa mag-boyfriend? … Nung pagtapos nun, hindi na lang ako umimik. … Hinayaan ko na lang kasi sabi ko [sa sarili ko], nangyari na nung una.  Helena, age 17 | The second time [my boyfriend forced me to have sex], I just thought, ‘We're approaching one year together already...’ I thought – because normally, it happens between a boyfriend-girlfriend couple, right? ... After that, I just didn't say anything. … I just let him do what he wanted because I thought, ‘It already happened the first time.’  Helena, age 17 |
